# Altered resting-state brain function in diabetic retinopathy: a meta-analysis of combined transcriptome-neuroimaging study

**DOI:** 10.3389/fendo.2026.1928717

**Published:** 2026-09-24

**Authors:** Xufeng Pu, Zhongru Sun, Weiqi Ji, Xiwei Yin, Yifan Li, Hongxia Zhang, Ji Zhang, Xiujuan Chen, Jianguo Xia

**Affiliations:** 1 Departments of Medical Imaging, The Affiliated Taizhou People’s Hospital of Nanjing Medical University, Taizhou, Jiangsu, China; 2 Departments of Ophthalmology, The Affiliated Taizhou People’s Hospital of Nanjing Medical University, Taizhou, Jiangsu, China

**Keywords:** diabetic cognitive impairment, diabetic encephalopathy, diabetic retinopathy, RS-fMRI, transcriptome-neuroimaging analysis

## Abstract

**Introduction:**

Patients with diabetes may develop cognitive dysfunction, particularly at advanced stages of the disease, manifested by impairments in learning, memory, language, and judgment. Previous studies have reported abnormal brain functional alterations in patients with diabetic retinopathy (DR), which may be associated with cognitive impairment in diabetes; however, the molecular correlates of these brain alterations remain poorly understood.

**Methods:**

In this study, we performed a coordinate-based neuroimaging meta-analysis to identify convergent resting-state brain functional alterations in patients with DR. By integrating the meta-analytic imaging phenotype with gene-expression data from the Allen Human Brain Atlas, we further conducted spatial transcriptomic -neuroimaging analyses to identify genes whose normative expression patterns were spatially associated with DR-related brain functional alterations.

**Results:**

The meta-analysis revealed relatively increased resting-state functional measures in the left cerebellum and corpus callosum, whereas relatively decreased measures were observed in the left calcarine fissure, left middle occipital gyrus, two clusters in the right postcentral gyrus, right anterior cingulate cortex, and right middle temporal gyrus. Functional decoding analyses indicated that these regions were associated with cognitive domains involving language, learning, and cognition. In addition, the regional neuroimaging phenotype was spatially associated with the normative expression patterns of 1,101 genes. Functional enrichment analyses indicated that these genes were enriched in processes related to learning, memory, and cognition, as well as biological processes involving metal ion regulation and cell adhesion. Cell- and tissue-specific enrichment analyses further suggested preferential expression patterns involving retinal and cortical neuronal populations.

**Discussion:**

Collectively, these findings demonstrate convergent resting-state brain functional alterations in patients with DR and reveal their spatial correspondence with normative gene-expression patterns, providing potential insights into the molecular and cellular context of cognitive-related brain alterations associated with DR.

**Systematic review registration:**

https://www.crd.york.ac.uk/prospero/, identifier CRD420261292056.

## Introduction

Diabetes Mellitus (DM) is a common chronic metabolic disease involving multiple systems. Diabetic Encephalopathy (DE) refers to the involvement of the central nervous system in diabetes, characterized by an insidious onset and slow progression, with pathological features and molecular mechanisms similar to late-onset Alzheimer’s dementia (LOAD) (1). Clinically, it manifests as decreased learning, memory, language expression and accompanied by symptoms such as cognitive impairment and dementia (2, 3). In recent years, cognitive impairment caused by diabetes has attracted increasing attention. Studies have shown that the rate of cognitive decline accelerates by approximately 68% within 4 years of new-onset diabetes (3), and the prevalence of cognitive impairment among patients with Type 2 diabetes mellitus (T2DM) is even as high as 32.8% (4), which has become a significant health issue of diabetic patients. According to data from the International Diabetes Federation (IDF), the global number of diabetic patients reaches 1.537 billion (5). With the extension of life expectancy in diabetic patients, the prevalence of DE has also increased correspondingly. Therefore, it is imperative to clarify the pathogenesis of DE and identify targets for its prevention and treatment.

Although the mechanism of Diabetic Encephalopathy (DE) remains unclear, a growing body of research suggests that microvascular dysfunction and impaired neurovascular coupling are closely interrelated factors contributing to this condition (6). Retinal and cerebral microvessels share embryological, morphological, and functional similarities, allowing retinal imaging to serve as a noninvasive tool for studying cerebral microvascular and neuronal damage (7, 8). This approach can assist in assessing dementia and stroke, supporting clinical diagnosis and prognosis (9–11). Diabetic retinopathy (DR), the most common microvascular complication of diabetes, often leads to severe visual impairment (12). Approximately 50% of patients develop DR within 10 years of diabetes diagnosis (13), and epidemiological projections indicate that by 2030, the global population affected by DR will reach 191 million (14). A number of longitudinal studies have confirmed a significant association between DR and DE, suggesting that cerebral functional changes in DR may represent early stages of DE. In the Edinburgh Type 2 Diabetes Study, increased severity of retinopathy was independently associated with cognitive decline (15). Another cohort study with a median follow-up of 4 years revealed that DR was positively correlated with a higher risk of mild cognitive impairment (16). In a further study by Exalto et al. involving 29,961 type 2 diabetes patients aged over 60, DR was associated with a 42% increased incidence of dementia (17). Consequently, some researchers propose retinal vascular density as a potential biomarker for cerebral small vessel disease in diabetes (18). These findings collectively indicate that diabetic retinopathy is a risk factor for the progression of DE, although its underlying molecular mechanisms still require further investigation.

Resting-state functional magnetic resonance imaging (rs-fMRI) is a non-invasive imaging technique used to detect brain function and metabolism by quantifying blood oxygen level-dependent (BOLD) signals and measuring local blood flow changes associated with neuronal activity (19, 20). Many research indicates that diabetic retinopathy (DR) is associated with significant alterations in brain function and structure. Results from the amplitude of low-frequency fluctuations (ALFF) analysis show reduced neuronal activity in the cerebellum, left cerebellum, left hippocampus, and bilateral middle occipital gyrus in DR patients (21–23). Arterial spin labeling (ASL) findings reveal decreased regional cerebral blood flow (CBF) in DR, suggesting insufficient perfusion in the visual cortex (24). Furthermore, functional connectivity (FC) studies demonstrate distinct FC patterns in the frontoparietal network (FPN), sensorimotor network (SMN), and visual network (VN) in the DR group (25). However, most studies remain focused on structural and functional changes in specific brain regions and have yet to elucidate their molecular characteristics.

In recent years, the refinement of the Allen Human Brain Atlas has made imaging genetics possible (26–29). The AHBA provides nearly 20,000 whole-brain genes. Under this framework, combining whole-brain gene expression data with brain imaging data helps identify gene expression profiles associated with patterns of structural and functional changes in the brain in psychiatric disorders (30–33). Early studies have already identified genes related to structural characteristics in the brains of psychiatric patients (32). This approach offers a research pathway from the macroscopic to the microscopic level. However, the spatial transcriptomic correlates of resting-state functional alterations in diabetic retinopathy (DR) remain largely unknown.

In the present study, we first conducted a comprehensive coordinate-based neuroimaging meta-analysis integrating multiple resting-state functional measures, including ALFF, ASL, VMHC, FC, and other related metrics, to identify convergent brain functional alterations in patients with DR. We then integrated the resulting meta-analytic imaging phenotype with gene-expression data from the Allen Human Brain Atlas (AHBA) and performed spatial transcriptomic–neuroimaging analyses to identify genes whose normative expression patterns were spatially associated with DR-related brain functional alterations. Finally, functional enrichment analyses were conducted to characterize the biological functions and pathways associated with these genes. An overview of the study workflow is presented in the graphical abstract.

## Methods

### Literature search and selection

This systematic review and coordinate-based neuroimaging meta-analysis was conducted and reported in accordance with the Preferred Reporting Items for Systematic Reviews and Meta-Analyses (PRISMA) 2020 statement (34). The review was registered in PROSPERO (registration No. CRD420261292056).

Two investigators independently searched PubMed and Web of Science from database inception to June 23rd, 2025, to identify studies examining resting-state brain functional alterations in patients with diabetic retinopathy (DR) compared with controls. The search strategy combined terms related to diabetic retinopathy with terms related to resting-state neuroimaging. The following keywords and their combinations were used: (“diabetic retinopathy”) and (“fMRI” or “functional magnetic resonance imaging” or “ALFF” or “amplitude of low-frequency fluctuations” or “dALFF” or “dynamic amplitude of low-frequency fluctuations” or “ReHo” or “regional homogeneity” or “ASL” or “arterial spin labeling” or “VMHC” or “voxel-mirrored homotopic connectivity” or “FC” or “functional connectivity”). In addition, the reference lists of relevant reviews, meta-analyses, and eligible original studies were manually screened to identify additional potentially eligible studies.

After duplicate records were removed, two investigators independently screened the titles and abstracts of all retrieved records. Potentially eligible studies were subsequently assessed through independent full-text review. Disagreements regarding study eligibility were resolved through discussion and consensus.

Studies were included if they met all of the following criteria: (1) original peer-reviewed studies published in English; (2) inclusion of patients with DR and an HC group; (3) investigation of resting-state brain functional differences between DR and HC using whole-brain voxel-wise neuroimaging analyses; and (4) reporting of statistically significant peak coordinates in Montreal Neurological Institute (MNI) or Talairach stereotactic space, together with sufficient statistical information for coordinate-based meta-analysis. Studies were excluded if they met any of the following criteria: (1) inclusion of participants of only one sex; (2) fewer than 10 participants in either the DR or HC group; or (3) animal studies. For the study by Qi et al., which reported DR vs DM, DR vs HC, and DM vs HC comparisons, only the DR vs HC contrast was included in the present meta-analysis to maintain consistency with the other included studies. The detailed study-selection process is presented in the PRISMA 2020 flow diagram (**Figure 1**), and a completed PRISMA 2020 checklist is provided in **Supplementary File S1**.

**Figure 1:**
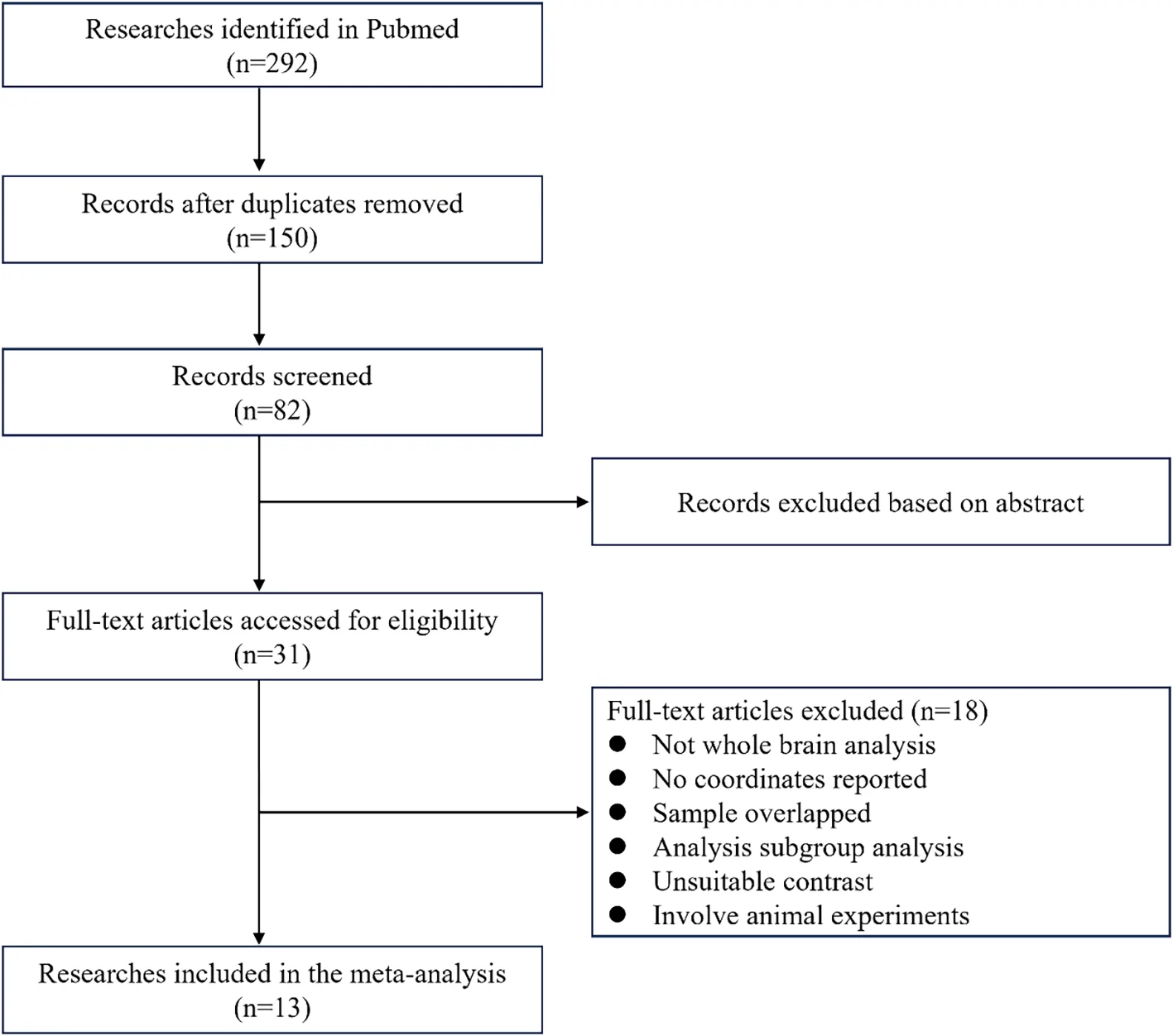
Flowchart of the study selection process.

### Data extraction

Two investigators independently extracted data from each eligible study using a standardized data-extraction form. The extracted information included the first author, participant age, sample size, sex distribution, duration of disease when available, resting-state imaging modality or metric, stereotactic coordinate space, direction of between-group differences, peak coordinates, and corresponding statistical values required for the SDM-PSI analysis. When required information was not available in the published article or **Supplementary Materials**, the study was excluded from the relevant analysis.

### Neuroimaging meta-analysis

A voxel-wise coordinate-based meta-analysis of resting-state functional alterations in DR was performed using Seed-based d Mapping with Permutation of Subject Images (SDM-PSI, version 6.23; https://www.sdmproject.com) (35). For each eligible study, peak coordinates and corresponding statistical values (e.g., t or z values) of significant between-group differences were extracted according to the SDM-PSI recommendations. Coordinates reported in Talairach space were converted to Montreal Neurological Institute (MNI) space using. Study-specific effect-size maps were reconstructed using the default SDM-PSI parameters, including a full width at half maximum of 20 mm and a voxel size of 2 mm. Effect-size estimation was performed using MetaNSUE, and pooled maps were generated using a random-effects model accounting for within-study variability and between-study heterogeneity. Results from multiple imputed datasets were combined according to Rubin’s rules. Positive SDM-Z values represented relatively increased resting-state functional measures in DR compared with HC, whereas negative values represented relatively decreased measures in DR compared with HC. Significant clusters were overlaid onto the MNI152 template using MRIcroGL and projected onto the cortical surface using BrainNet Viewer. To evaluate the reliability and robustness of the meta-analytic findings, several supplementary analyses were performed. Between-study heterogeneity was assessed using the I² statistic. Potential small-study effects were evaluated using Egger’s regression test and Begg’s rank correlation test, with P values adjusted for multiple comparisons using the Holm method. Whole-brain jackknife sensitivity analyses were conducted by sequentially omitting one study at a time and repeating the meta-analysis. In addition, leave-one-modality-out sensitivity analyses were performed to assess whether the pooled findings were disproportionately driven by a specific resting-state imaging measure. In each iteration, all studies using one imaging modality were excluded and the SDM-PSI meta-analysis was repeated using the same analytical settings as the primary analysis. A principal finding was considered preserved when a significant cluster with the same effect direction remained within the same anatomical region; findings showing modest spatial displacement of the peak but preserved anatomical location and effect direction were also considered retained (36, 37).

### Behavioral relevance analysis

BrainMap is a database with published neuroimaging experiments on tasks and structures, which contains coordinate-based experimental results (http://www.brainmap.org/). The internal plugins in Mango were used to determine behavioral relevance of related resting-state brain functional alterations in DR, and the specific methods can be found in the plugin introduction.

### Brain gene expression and correlated to resting-state brain functional alterations in DR

Brain-wide gene-expression data were obtained from the Allen Human Brain Atlas (AHBA), which includes microarray expression measurements from six postmortem human donors (26). Gene-expression data were processed using abagen v0.1.3 and the Brainnetome Atlas (BNA-246) (https://www.github.com/netneurolab/abagen). Probe-to-gene annotations were updated using the Re-Annotator procedure, probes that did not exceed background noise in at least 50% of samples across donors were excluded, and, where multiple probes mapped to the same gene, the probe showing the highest correspondence with RNA-seq expression was selected. Tissue samples were assigned to atlas regions according to their spatial coordinates, and expression values were normalized using a scaled robust sigmoid procedure (38). Genes were further filtered according to differential stability. Because of the sparse right-hemisphere sampling in the AHBA, left–right mirroring was applied during preprocessing to obtain bilateral regional expression profiles, and missing regional expression values were interpolated where necessary.

To construct the regional imaging phenotype, the unthresholded whole-brain SDM-Z map was parcellated according to the BNA-246 atlas, and the mean signed SDM-Z value was calculated for each of the 246 regions. All regions were retained without significance-based filtering; thus, regions without statistically significant meta-analytic effects retained their continuous signed SDM-Z values rather than being excluded or set to zero. PLS regression was then performed using the regional gene-expression matrix as the predictor matrix and regional signed SDM-Z values as the response variable. Both predictors and response values were z-standardized before analysis. Two PLS components were estimated using MATLAB plsregress, and PLS1 was used to characterize the dominant spatial covariance between gene expression and the imaging phenotype. Statistical significance was assessed using Moran spectral randomization with 5,000 permutations to account for spatial autocorrelation. The stability of individual PLS1 gene weights was further assessed using 5,000 bootstrap resamples, and a Z score was calculated for each gene by dividing its PLS1 weight by the corresponding bootstrap-estimated standard error (39). Genes satisfying |Z| > 3 and FDR-corrected q < 0.05 were retained, yielding 1,101 genes for downstream analyses.

### Gene enrichment and protein-protein interaction analysis

The XianTao (https://www.xiantaozi.com/) was used to perform enrichment, including Gene ontology (GO), Kyoto Encyclopedia of Genes and Genomes (KEGG), and both select top 10 results to plot. The Web-based Cell-type-Specific Enrichment Analysis of Genes (https://bioinfo.uth.edu/webcsea/) was used to determine the organ, tissue, and cell specific expression of resting-state brain functional alterations related genes. The specific expression enrichment of brain region and temporal-specific expression analysis were conducted by (http://doughertytools.wustl.edu/CSEAtool.html).

Protein–protein interaction (PPI) networks were constructed using the stringApp plugin in Cytoscape, with the minimum required interaction score set to 0.9, corresponding to the highest-confidence interaction threshold. Network centrality was subsequently assessed using the CytoNCA plugin. Genes were ranked according to node degree, and those within the top 10% of the degree distribution were defined as hub genes. The spatial and temporal expression patterns of representative hub genes were then examined using the Human Brain Transcriptome Atlas (https://hbatlas.org/).

### Spatial correlation between resting-state brain function alterations with neurotransmitter systems, excitatory neurons and mitochondria function profiles

Spatial correlations with neurotransmitter systems, excitatory neurons and mitochondria function profiles were calculated using the JuSpace toolbox (https://github.com/juryxy/JuSpace). We considered Neurotransmitter profile including dopamine receptor family (D1 receptors, D2 receptors), dopamine transporter (DAT), 5‐HT1a receptors, 5‐HT1b receptors, 5‐HT2a receptors, 5‐HT4 receptors, 5‐HT6 receptors, serotonin transporter (SERT), γ-aminobutyric acid receptor (GABA), kappa opioid receptor (KappaOp), Muscarinic acetylcholine receptor (M1), Synaptic Vesicle Protein 2A (SV2A), Histone Deacetylase (HDAC). Excitatory neurons profile including Ex1-CortProject-L23, Ex2-Granule-L34, Ex3-Granule-L4, Ex4-SubcortProject-L4, Ex5-SubcortProject-L456, Ex6-SubcortProject-L56, Ex7-CorticothalProject, Ex8-CorticothalProject-L6. Mitochondria Profiles including Complex1-NADH, Complex2-SDH, Complex-COX, Respiratory Capacity, cerebral metabolic rate of glucose (CMRglu). The p‐values were considered significant at FDR‐corrected p < 0.05.

## Results

### Brain regions and behavior analysis with resting-state brain functional alterations between DR and HC

Our meta-analysis included 13 studies comprising 582 patients with DR and 573 healthy controls (HCs). The clinical and imaging characteristics of the included studies are summarized in **Table 1**. Compared with HCs, patients with DR showed relatively increased resting-state functional measures in the left cerebellum and corpus callosum, whereas relatively decreased measures were observed in the left calcarine fissure, left middle occipital gyrus, right postcentral gyrus, right anterior cingulate cortex, and right middle temporal gyrus (**Table 2**; **Figures 2A, B**). Between-study heterogeneity was generally low, with I² values ranging from 0.8% to 29.8%. After Holm correction, significant Egger asymmetry was observed only for one right postcentral gyrus cluster (adjusted P = 0.013), suggesting a potential small-study effect, whereas all other Egger and Begg tests were non-significant (**Supplementary Table S1**). Jackknife and leave-one-modality-out sensitivity analyses showed that the principal findings were generally robust, although the corpus callosum, right middle temporal gyrus, and left cerebellar findings showed relatively greater sensitivity (**Supplementary Tables S2**–**S5**).

**Table 1:** The clinical and imaging characteristics of studies.

Study	Number/female		Age		Duration of disease (years)	fMRI
	DR	HC	DR	HC	DR	Measures
Shi et al. (22)	24/14	24/14	54.31 ± 5.96	53.08 ± 5.33	0.69 ± 0.20	ALFF
Wang et al. (23)	21/11	16/9	54.9 ± 9.9	54.8 ± 5.70	9.48 ± 5.01	ALFF
Qi et al. (21)	35/17	38/20	54.17 ± 8.67	53.47 ± 7.73	9.94 ± 5.10	ALFF
Zhong et al. (24)	31/10	31/10	51.16 ± 1.23	52.15 ± 2.21	N/A	ASL
Wang et al. (40)	20/11	20/11	53.94 ± 8.65	55.67 ± 9.25	12.61 ± 5.71	DC
Huang et al. (41)	46/24	43/21	N/A	N/A	N/A	VMHC
Wang et al. (42)	22/12	22/12	55.27 ± 7.25	54.80 ± 6.10	10.20 ± 6.28	VMHC
Zhong et al. (43)	47/21	46/22	54.84 ± 3.22	55.36 ± 4.48	11.54 ± 4.37	dFCD
Liao et al. (44)	28/16	28/16	57.22 ± 5.18	56.81 ± 5.06	0.66 ± 0.20	Reho
Li et al. (45)	21/11	21/11	55.9 ± 9.7	55.4 ± 9.00	N/A	FC
Qi et al. (46)	35/17	32/12	53.37 ± 7.56	54.46 ± 8.06	5.52 ± 3.96	FC
Yu et al. (47)	26/14	26/14	55.53 ± 5.83	54.34 ± 5.79	0.72 ± 0.19	FC
Chen et al. (48)	34/14	37/17	45.80 ± 2.46	46.00 ± 2.68	N/A	FC

DR, patients with diabetic retinopathy; HC, healthy controls; NA, not available; fMRI, functional magnetic resonance; ALFF, amplitude of low frequency fluctuations; ASL, arterial spin labeling; DC, degree centrality; VMHC, voxel-mirrored homologous connection; dFCD, dynamic functional connectivity density; Reho, regional homogeneity; FC, functional connectivity.

**Table 2:** The functional alterations between DR and HC identified by voxels meta-analysis.

Regions	MMI coordinate	Voxels	SDM-Z	P-value
DR>HC				
Left cerebellum	-14, -74, -42	468	3.678	1.17×10^-4^
Corpus callosum	14, -66, 40	101	3.543	1.97×10^-4^
DR<HC				
Left calcarine fissure	-2, -68, 14	2879	-6.396	<0.0001
Left middle occipital gyrus	-20, -98, 4	1444	-8.026	<0.0001
Right postcentral gyrus	62, -14, 18	1966	-6.324	<0.0001
Right anterior cingulate	4, 44, 4	1853	-7.133	<0.0001
Right postcentral gyrus	32, -34, 54	1469	-5.492	<0.0001
Right middle temporal gyrus	54, -8, -24	79	-2.467	0.0068

DR, patients with diabetic retinopathy; HC, healthy controls; MNI, Montreal Neurological Institute; SDM, seed-based d mapping.

**Figure 2:**
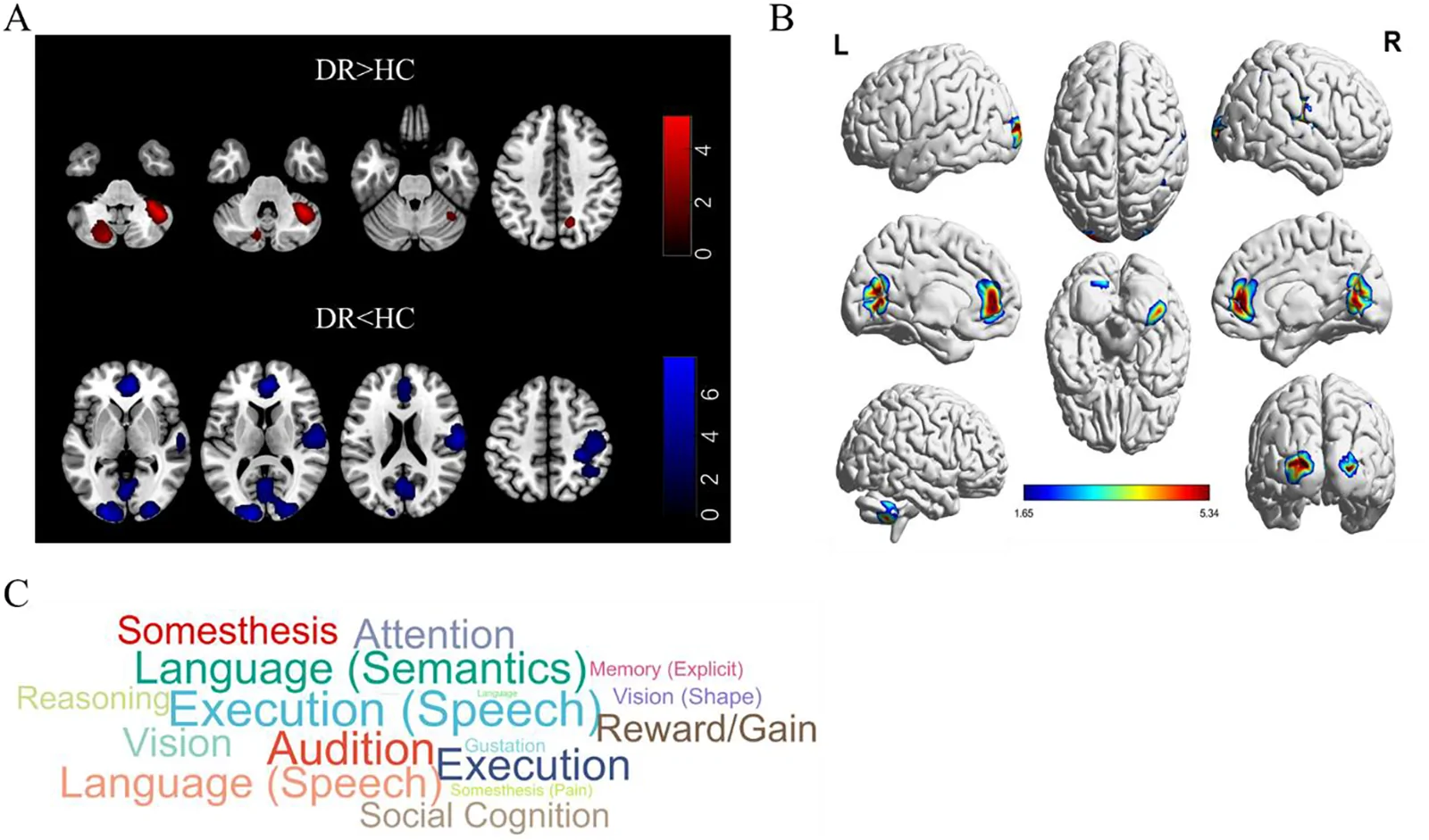
Brain regions and behavior analysis with resting-state brain functional alterations between DR and HC. **(A, B)** Resting-state brain functional alterations in patients with DR, the red and blue color in figure **(A)** represents significantly increased and decreased voxels. The color bar in figure **(B)** represents significance of brain regions alterations. **(C)** The behavior analysis of brain regions with functional alterations. The size of words represents Z-score.

To determine the function of brain regions, we performed behavior analysis through Mango plug and these regions related to execution (speech), audition, language(semantics), language(speech), attention, vision, somesthesis, reward/gain, social cognition, reasoning, vision(shape), memory(explicit), gustation, language(orthography) (**Figure 2C**).

### Enrichment analysis of genes associated with resting-state brain functional alterations in DR

1101 genes were determined to be associated with brain functional alterations in DR through transcription-neuroimaging spatial correlation analysis. To elucidate the functional of these genes and related pathways, we performed enrichment analysis. The KEGG results reveled top 10 pathways, such as focal adhesion, proteoglycans in cancer, ECM-receptor interaction, etc. (**Figure 3A**; **Supplementary File 1**). Additionally, the Go analysis of biological process was identified, including cognition, modulation of synaptic transmission, regulation trans-synaptic signaling, learning or memory, regulation of neurotransmitter receptor activity, response to calcium ion, developmental cell growth, cellular response to metal ion, and development growth involved in morphogenesis (**Figure 3B**; **Supplementary File 2**). Moreover, top 10 cellular components were annotated, such as glutamatergic synapse, postsynaptic density, neuron to neuron synapse, asymmetric synapse, etc. (**Figure 3C**; **Supplementary File 3**). In terms of molecular functions, these genes related to profilin binding, actin binding, phosphatidylinositol phosphate binding, GTPase regulator activity, etc. (**Figure 3D**; **Supplementary File 4**).

**Figure 3:**
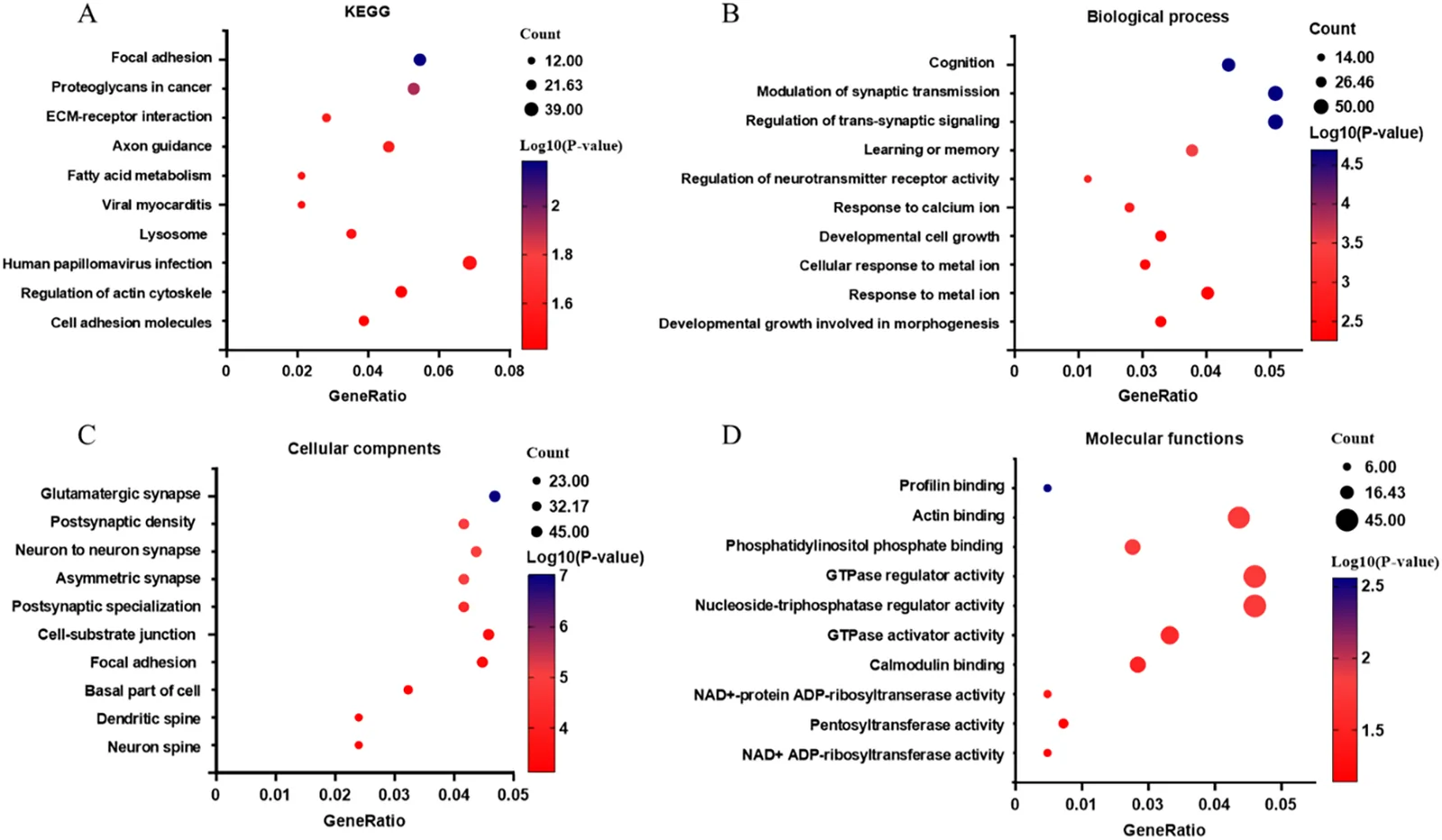
Enrichment analysis of genes in resting-state brain functional module. **(A)** KEGG, Kyoto Encyclopedia of Genes and Genomes. **(B)** Go: Biological process. **(C)** Go: Cellular component. **(D)** Go: Molecular function. The x-axis reflects enrichment score and the y-axis shows items. The color of bubble shows the statistical significance and the bubble size represents the number of genes belonged to item respectively.

For the 1101 genes associated with DR, the cell-type-specific enrichment analysis (CSEA) of organ results revealed that these genes mainly enriched in digestive system, lymphatic system and nervous system (**Figure 4A**). Additionally, in terms of tissue and general cells, these genes mainly enriched in eye, pancreas, visual cortex, frontal cortex, endothelial cell, excitatory neuron, capillary endothelial cell, epithelial cell and pericyte cell (**Figures 4A–C**). Moreover, to further known the cell function, we performed the brain cell type expression and the results indicated that these genes specifically expressed in Ctx (Cortex), Ctx.Pdgfrajd340 (Oligodendrocyte Progenitor Cells of cortex), Ctx.Ntsr (Ntsr+ neurons of cortex), Ctx.Cort (Cort+ neurons of cortex) and Ctx.Cnp (Myelinating Oligodendrocyte of cortex) (**Figure 4D**). The temporal-specific expression further revealed that these genes are preferentially expressed in cortex and thalamus in the stage of young adulthood (**Figures 4E, F**).

**Figure 4:**
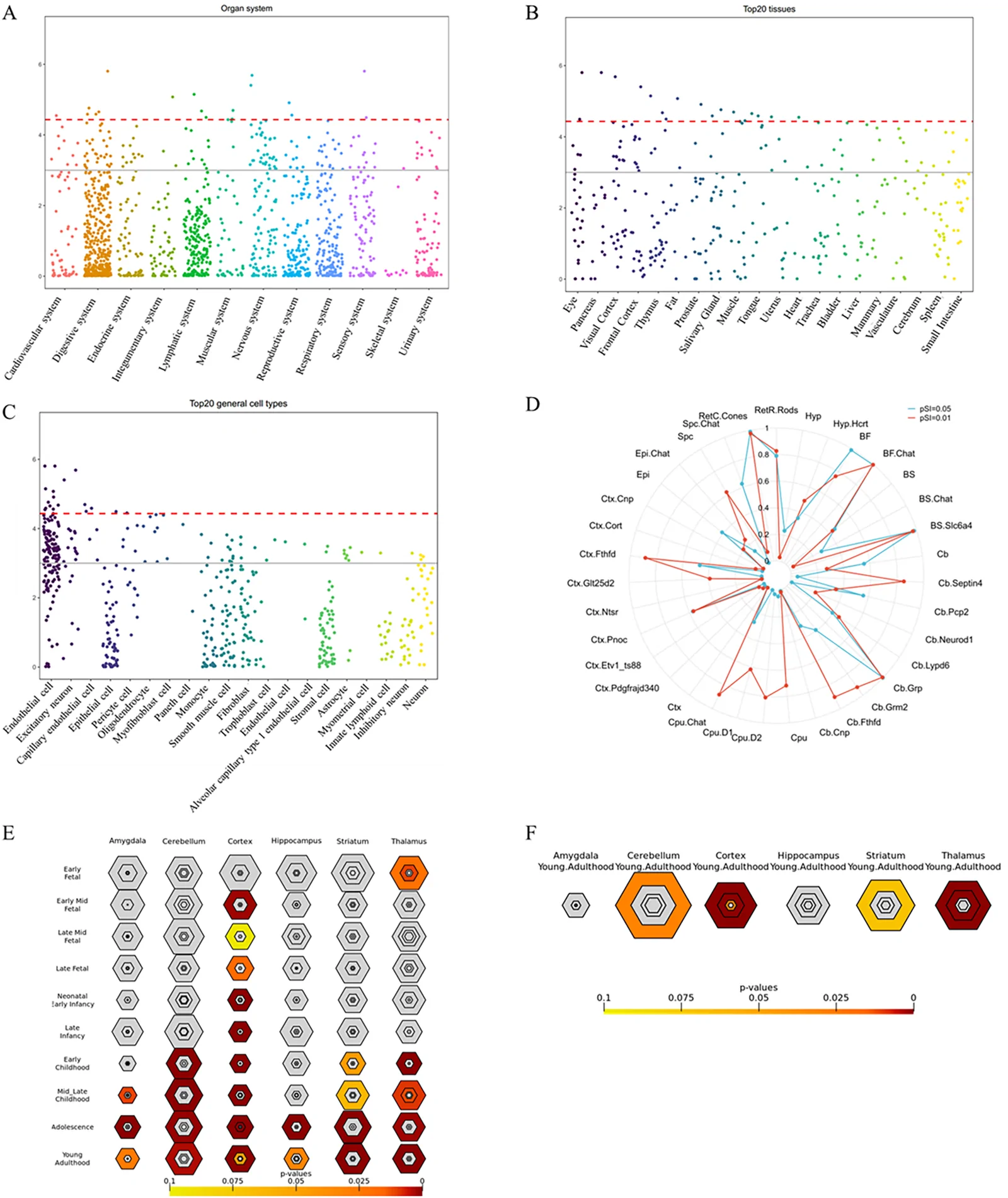
The cell-type-specific enrichment analysis of DR related genes. **(A–C)** The specific expression pattern in organ, tissue and cell. The red dashed line indicates the Bonferroni-corrected significance (p = 3.69 x 10^-5). The grey solid line indicates the nominal significance (p = 1 x 10^-3). **(D)** The cell-specific expression in brain. The blue and red color in radar plot represents the result at pSI=0.05 and pSI=0.01, respectively. **(E, F)** Specific expression analysis across brain regions and development of genes.

### PPI network analysis and hub genes

To know the interaction of genes, we used Cytoscape to construct PPI networks and identified top 10 genes (**Figures 5A, B**). Additionally, we delineated the spatial-temporal expression patterns of three most representative genes, including CALML4, CALML5, and CALML6 (**Figures 5C–E**).

**Figure 5:**
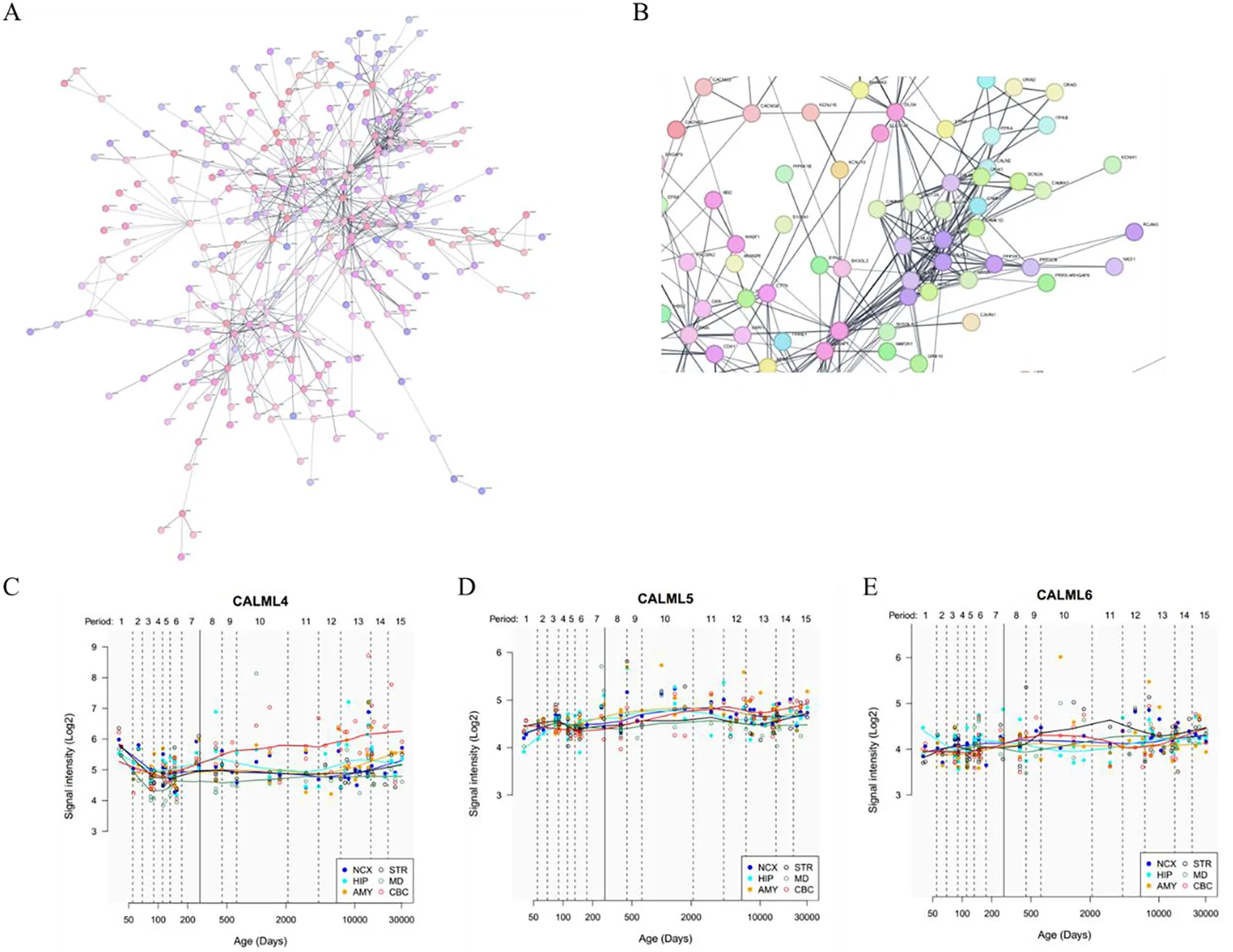
PPI network and hub genes. **(A, B)** The PPI network with resting-state brain associated genes. **(C)** The spatial and temporal expression curves of three hub genes(CALML4, CALML5 and CALML6).

### Spatial correlation with neurotransmitter systems, mitochondria function profiles and excitatory neurons.

We correlated the resting-state brain functional alterations with neurotransmitter, mitochondria function and excitatory neurons, according to the enrichment results. The resting-state brain functional alterations were significantly correlated 5HT2a (*ρ* =0.3425, *p* FDR = 0.0009), 5HT6 (*ρ* =0.2075, *p* FDR = 0.0259), SERT (*ρ* =-0.1919, *p* FDR = 0.0329), HDAC (*ρ* =0.3482, *p* FDR = 0.0009), Complex-2-SDH (*ρ* =0.3781, *p* FDR = 0.0029), CMRglu (*ρ* =0.2352, *p* FDR = 0.0109), EX3-Granule-L4 (*ρ* =0.5503, *p* FDR = 0.0009) (**Figures 6A–C**).

**Figure 6:**
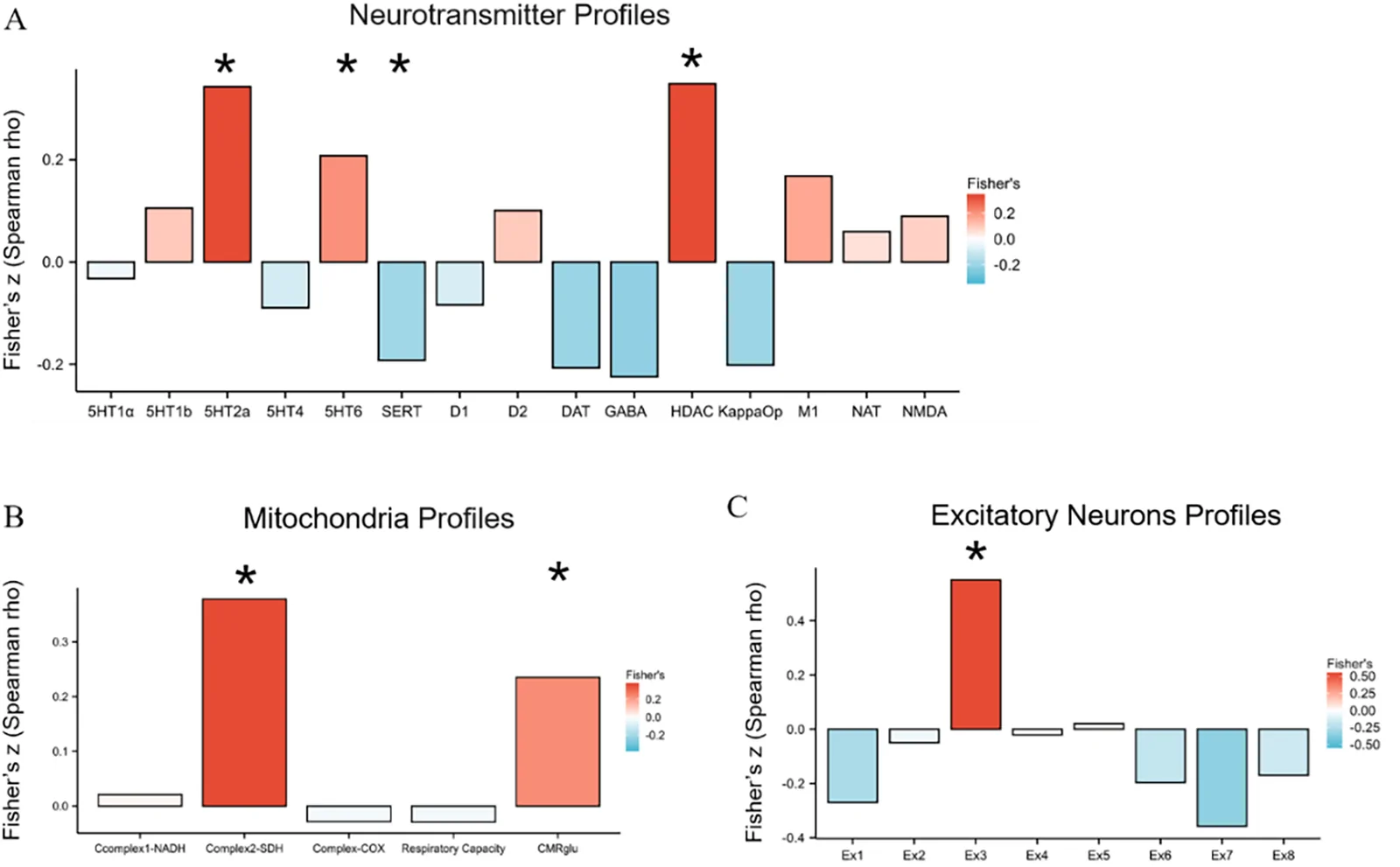
Neurotransmitter system, mitochondrial and excitatory neurons profiles related to resting-state brain alterations. **(A)** Correlation between resting-state brain alterations and neurotransmitter profiles. 5-HT1a, 5‐HT subtype1a; 5‐HT1b, 5‐HT subtype1b; 5‐HT2a, 5‐HT subtype2a; 5HT4, 5‐HT subtype4; 5HT6, 5‐HT subtype6; SERT, serotonin transporter; D1, D1 receptors; D2, D2 receptors; DAT, dopamine transporter; GABA, γ-aminobutyric acid receptor; HDAC, Histone Deacetylase; KappaOp, kappa opioid receptor; M1, Muscarinic acetylcholine receptor; NAT, Noradrenaline Transporter; NMDA, N-methyl-D-aspartic acid receptor; **(B)** Correlation between resting-state brain alterations and mitochondrial function. NADH, Nicotinamide adenine dinucleotide; SDH, Succinate dehydrogenase; COX, Cytochrome c Oxidase; CMRglu, cerebral metabolic rate of glucose; **(C)** Correlation between dReHo alterations and excitatory neurons. Ex1, Ex1-CortProject-L23; Ex2, Ex2-Granule-L34; Ex3, Ex3-Granule-L4; Ex4, Ex4-SubcortProject-L4; Ex5, Ex5-SubcortProject-L456; Ex6, Ex6-SubcortProject-L56; Ex7, Ex7-CorticothalProject; Ex8, Ex8-CorticothalProject-L6. * indicates statistical significance (P < 0.05).

## Discussion

Diabetic retinopathy (DR) is a common microvascular complication of diabetes and has been increasingly associated with cognitive dysfunction. In the present study, we combined a coordinate-based neuroimaging meta-analysis with Allen Human Brain Atlas (AHBA) data to investigate the spatial molecular correlates of brain functional alterations in DR. Convergent abnormalities were identified in visual, sensorimotor, cingulate, cerebellar, callosal, and temporal regions, which were functionally related to language, vision, memory, attention, and social cognition. Imaging-transcriptomic analysis further identified 1,101 genes whose normative expression patterns were spatially associated with the regional neuroimaging phenotype and enriched in neural signaling, cognition, learning, and memory-related processes. These findings provide new insight into the molecular context of DR-associated brain dysfunction.

Our neuroimaging and CSEA findings converge on brain regions that may be relevant to diabetes-related cognitive dysfunction. Patients with DR showed relatively increased resting-state functional measures in the left cerebellum and corpus callosum, while genes spatially associated with the imaging phenotype showed enrichment in the retina, occipital cortex, frontal cortex, and cerebellum. Although the cerebellum has traditionally been regarded as a center for motor control, increasing evidence supports its involvement in higher-order cognitive functions. Cerebellar abnormalities have been reported in Alzheimer’s disease and diabetes and have been associated with cognitive impairment, reduced neuronal activity, and disrupted functional connectivity with frontoparietal and visual networks (49, 50). The corpus callosum, the major white matter tract connecting the two cerebral hemispheres, is essential for interhemispheric information transfer and integration (51) (52). Previous studies have also reported altered cerebellar and callosal connectivity in DR (58), while experimental evidence suggests that pathological changes in the corpus callosum may contribute to diabetes-related cognitive impairment (53). Although the Mango-based functional decoding did not identify specific behavioral terms for the cerebellum or corpus callosum, the converging evidence suggests that these regions may represent important components of the neural vulnerability associated with DR and diabetes-related cognitive dysfunction. Further studies are needed to clarify their specific contribution, particularly given that the cerebellum is not consistently included in neuroimaging analyses.

The calcarine fissure and middle occipital gyrus are located in the occipital cortex and play important roles in visual processing and attention. Previous studies have reported reduced cerebral blood flow around the calcarine fissure and decreased centrality in the middle occipital gyrus in patients with DR (24, 54). Beyond visual processing, the calcarine cortex may also participate in cross-modal language processing under conditions of visual deprivation (55, 56). Clinically, cognitive impairment in diabetes manifests primarily as declines in learning ability, memory, language expression, and judgment (57). Our behavioral analysis indicates that the calcarine fissure in DR patients is significantly associated with language and visual functions. Additionally, in diabetic retinopathy, the middle occipital gyrus, as a key node of the visual network, shows weakened hub properties and exhibits abnormal functional connectivity with the visual cortex and other higher-order cortical regions (47, 54). Meanwhile, brain regions closely related to the function of the middle occipital gyrus, such as the precuneus, also demonstrate significant spontaneous neural activity abnormalities and structural alterations (58, 59). Together, these findings suggest that alterations in the calcarine fissure and middle occipital gyrus may contribute to visual–cognitive dysfunction and functional reorganization of the central nervous system in DR.

The middle temporal gyrus and cingulate cortex are important components of the default mode network (DMN) and related large-scale cognitive networks (60). Previous studies have reported reduced interhemispheric functional connectivity of the bilateral middle temporal gyrus and altered resting-state cerebral blood flow in the middle temporal region in patients with DR (61, 62). By contrast, the role of the cingulate cortex in DR remains less well characterized. In patients with type 2 diabetes and early cognitive impairment, preserved cingulate cortical thickness has been observed despite reduced functional connectivity with the bilateral hippocampi (63). Together with our findings, these observations suggest that functional abnormalities involving the middle temporal and cingulate regions may emerge before overt structural changes become detectable. Further studies are needed to determine whether these alterations contribute to the progression of cognitive dysfunction in DR.

Imaging-transcriptomic spatial analysis identified genes whose normative expression patterns were associated with the DR-related resting-state imaging phenotype. Functional enrichment analyses indicated that these genes were mainly involved in cell adhesion, neurotransmission, synaptic function, and ion-channel-related processes, with KEGG analysis further highlighting cell adhesion pathways. Cell adhesion has an established role in the vascular pathology of DR. Leukocyte–endothelial adhesion is considered an early event in DR, whereby activated leukocytes adhere to the retinal vascular endothelium and may contribute to capillary occlusion and ischemia (64). Consistently, circulating soluble vascular cell adhesion molecule-1 (sVCAM-1) levels have been reported to be elevated in patients with proliferative diabetic retinopathy (PDR) (65). Extracellular matrix-related proteins may also participate in this process; for example, vitronectin, an integrin-binding glycoprotein, is elevated in the vitreous and plasma of patients with PDR and has been associated with blood–retinal barrier disruption, neovascularization, and coagulation abnormalities (66). Given the shared neurovascular characteristics of the retina and brain, these observations raise the possibility that cell-adhesion-related processes may represent part of the molecular context associated with brain functional alterations in DR. However, this interpretation remains hypothesis-generating and requires direct experimental validation.

Cognitive impairment associated with diabetes commonly involves deficits in learning, memory, language, and judgment (57), and previous studies have suggested a close association between DR and cognitive dysfunction (13, 14). In our study, genes whose normative expression patterns were spatially associated with the DR-related imaging phenotype were enriched in biological processes related to cognition, learning, memory, ion regulation, and synaptic function. These findings provide a potential molecular context for the cognitive-related brain abnormalities observed in DR, although they should not be interpreted as evidence that these genes are altered in patients with DR. Metal-ion homeostasis and synaptic function may be particularly relevant to this process. Dysregulation of metal ions has been implicated in oxidative stress and neurodegenerative processes, including protein aggregation and reactive oxygen species generation (67). Under hyperglycemic conditions, such disturbances may further aggravate oxidative injury in retinal and neural tissues. Synaptic function is also closely coupled to neurovascular regulation. Retinal microglia interact with both blood vessels and neural synapses and may contribute to neurovascular coupling under physiological conditions (68, 69). In diabetes, microglial activation and metabolic disturbances may disrupt this interaction, thereby impairing vascular responses to neural activity (68). Together, these observations suggest that altered ion homeostasis, oxidative stress, and synaptic dysfunction may represent interconnected biological processes relevant to DR-associated neural abnormalities. Glutathione-related antioxidant dysfunction may provide an additional link between metabolic stress and neural injury. Reduced glutathione levels have been reported in the cerebral cortex, cerebellum, brainstem, and hippocampus of diabetic animal models (70–73). As glutathione is a major component of the cellular antioxidant system and is closely related to ferroptosis (74), impairment of glutathione homeostasis may increase susceptibility to oxidative and iron-dependent neuronal injury (75, 76). However, these mechanisms remain speculative in the context of our imaging-transcriptomic findings and require direct validation in patients with DR.

Among the representative hub genes, CALML4, CALML5, and CALML6 belong to the calmodulin-like family and may participate in calcium-dependent signaling. Although direct evidence linking these genes to DR remains limited, previous studies suggest potential relevance to ocular and neurological processes. CALML5 has been reported as an upregulated tear-exosomal protein in dry eye disease (77), while CALML4 shows altered expression in experimental models of depression and has been implicated in regulatory networks involving neurotrophic, MAPK, PI3K-Akt, cAMP, GABAergic, and circadian pathways (78). Notably, GeneCards annotations indicate that all three genes are related to the renin–angiotensin–aldosterone system (RAAS), which is strongly implicated in diabetes, diabetic microvascular complications, and central nervous system regulation. Excessive activation of the classical RAAS can promote oxidative stress, endothelial dysfunction, inflammation, and microvascular injury, mechanisms that are highly relevant to retinal vascular damage in DR (79, 80); meanwhile, an intrinsic brain RAAS contributes to cerebrovascular regulation, neuroinflammation, blood–brain barrier integrity, and cognitive function (80). These observations raise the possibility that CALML4, CALML5, and CALML6 may lie at the intersection of calcium/calmodulin-related signaling and RAAS-associated regulation, potentially linking retinal microvascular abnormalities with cerebral functional and cognitive changes in individuals with DR. Future studies should determine whether these genes modulate retinal and cognitive phenotypes through RAAS-related mechanisms in diabetic and DR models.

Our spatial correlation analyses further showed that the DR-related resting-state imaging phenotype was spatially associated with markers of the serotonergic system, including 5-HT2A, 5-HT6, and SERT, as well as indices related to cerebral glucose metabolism, including Complex II/succinate dehydrogenase (SDH) and CMRglu, and the EX3-Granule-L4 cortical cell type. Impaired cerebral glucose utilization has been reported in diabetes-related cognitive impairment and may contribute to regional hypometabolism and neuronal dysfunction (81, 82). SDH is a key enzyme of both the tricarboxylic acid cycle and mitochondrial respiratory Complex II, linking cellular metabolism with mitochondrial energy production (83). Disturbance of SDH-related mitochondrial function may reduce ATP production and increase oxidative stress, potentially affecting synaptic and neuronal integrity. The serotonergic system also plays an important role in cognition and behavior. In particular, 5-HT2A and 5-HT6 receptors have been implicated in working memory, cognitive flexibility, learning, memory, and neuroplasticity, whereas SERT regulates the duration and intensity of serotonergic neurotransmission through presynaptic serotonin reuptake (84–87). In addition, the association with EX3-Granule-L4 is noteworthy because layer IV cortical neurons are major recipients of thalamocortical sensory input. Although their role in diabetic cognitive impairment remains unclear, studies in neurodegenerative disorders have linked layer IV neuronal abnormalities to synaptic maintenance, plasticity, calcium homeostasis, and neuroprotection (88). Together, these findings suggest that regional brain functional alterations in DR may occur within a molecular context involving energy metabolism, serotonergic neurotransmission, and specific cortical neuronal populations. However, these spatial associations are exploratory and warrant further validation in future studies.

## Limitations

Several limitations should be acknowledged. First, gene-expression data were derived from postmortem healthy donors in the AHBA, whereas the neuroimaging data were obtained from living individuals with DR, which may limit the correspondence between normative spatial expression patterns and disease-related molecular alterations. Second, the sparse right-hemisphere sampling in the AHBA may introduce hemispheric bias despite bilateral preprocessing. Third, the meta-analysis combined heterogeneous resting-state imaging measures, and although sensitivity analyses supported the overall robustness of the main findings, these metrics are not biologically equivalent. Fourth, all contrasts compared patients with DR with non-diabetic healthy controls, so the effects of diabetes itself cannot be disentangled from retinopathy-related alterations. Finally, the relatively small number of eligible studies may also affect the robustness and generalizability of the findings. Further validation using patient-derived transcriptomic data and experimental models is warranted.

## Conclusion

In summary, our meta-analysis identified convergent resting-state brain functional alterations across eight regions in patients with DR, involving networks related to visual processing, cognition, and other higher-order functions. Imaging-transcriptomic analysis further revealed spatial associations between these regional abnormalities and normative gene-expression patterns, with enrichment in the visual cortex, frontal cortex, and cerebellum and in biological processes related to neural signaling and cognition. These findings provide a molecular and cellular context for DR-associated brain dysfunction and offer new insights into the neurobiological features of DR. Further studies using patient-derived molecular data and experimental models are needed to validate these associations and clarify their potential clinical relevance.

## Data Availability

The original contributions presented in the study are included in the article/**Supplementary material**. Further inquiries can be directed to the corresponding authors.
