# Supplementary material for: Altered resting-state brain function in diabetic retinopathy: a meta-analysis of combined transcriptome-neuroimaging study
Source: Front Endocrinol (Lausanne). 2026 Sep 24;17:1928717. doi: 10.3389/fendo.2026.1928717 (PMC13645568; doi:10.3389/fendo.2026.1928717)
Supplement: Supplementary file 1 [file SupplementaryFile1.docx]

**Supplementary Material**

**Altered resting-state brain function in Diabetic retinopathy: A meta-analysis of combined transcriptome-neuroimaging study**

**Xufeng Pu^a^** **^1^, Zhongru Sun^a^** **^1^, Weiqi Ji^a^** **^1^, Xiwei Yin^a^** **^1^, Yifan Li^a^, Hongxia Zhang^a^, Ji Zhang, Xiujuan Chen^b*^,** **Jianguo Xia^a *^**

**Affiliations**

^a^ Departments of Medical Imaging, The Affiliated Taizhou People’s Hospital of Nanjing Medical University, Taizhou, Jiangsu, China.

^b^ Departments of Ophthalmology, The Affiliated Taizhou People’s Hospital of Nanjing Medical University, Taizhou, Jiangsu, China.

^1^These authors contributed equally to this work.

* Corresponding author at the Affiliated Taizhou People’s Hospital of Nanjing Medical University, 36 Taihu Road, Fenghuang Subdistrict, Gaogang District, Taizhou City, Jiangsu Province

E-mail address: xiajianguo@njmu.edu.cn (J. Xia); 740275214@qq.com

(X. Chen); zj00151@126.com (J. Zhang)

Contents

[Supplementary methods	3](#_Toc239582839)

[Literature search and selection	3](#_Toc239582840)

[Transcription-neuroimaging association analysis	3](#_Toc239582841)

[Gene enrichment analysis	4](#_Toc239582842)

[Supplementary Tables	5](#_Toc239582843)

[Supplementary Figures	9](#_Toc239582844)

[Figure S1. Funnel plots for the principal meta-analytic findings.	9](#_Toc239582845)

[Supplementary references	11](#_Toc239582846)

# Supplementary methods

## Literature search and selection

The methodological quality of the included studies was assessed using a 10-point checklist that has been widely used in previous neuroimaging meta-analyses(Baiano, et al. 2007; Cai, et al. 2024). The checklist covers three main domains: participant demographic and clinical characteristics, image acquisition and analysis methods, and the reporting of results and conclusions. Each item was assigned a score of 1, 0.5, or 0, corresponding to fully met, partially met, or not met, respectively. Studies were classified as low quality (<4), moderate quality (5–7), or high quality (≥8) according to the total score(Medisauskaite and Kamau 2017). The assessed items included: (1) clear diagnostic criteria for diabetic retinopathy; (2) adequate reporting of demographic characteristics; (3) adequate reporting of relevant clinical characteristics; (4) appropriate definition of the control group and group comparability; (5) adequate description of MRI acquisition parameters; (6) adequate description of image preprocessing procedures; (7) appropriate control of head motion; (8) whole-brain voxel-wise statistical analysis with clearly reported statistical thresholds; (9) adequate reporting of stereotactic coordinates and corresponding statistical values; and (10) clear presentation of results and conclusions supported by the reported findings.

## Transcription-neuroimaging association analysis

Brain-wide gene-expression data were obtained from the Allen Human Brain Atlas (AHBA), which contains microarray expression measurements from six postmortem donors. Data were preprocessed using *abagen* (version 0.1.3) and parcellated according to the Brainnetome Atlas (BNA-246) (Albajes-Eizagirre, et al. 2019). First, microarray probes were reannotated to genes using the Re-Annotator procedure. Probes whose expression did not exceed background noise in at least 50% of samples across donors were excluded. For genes represented by multiple probes, the probe showing the highest correspondence with RNA-seq expression was selected. Tissue samples were assigned to BNA parcels according to their MNI coordinates, using a 2-mm Euclidean distance criterion for parcel assignment. Expression values were normalized within each donor using a scaled robust sigmoid transformation. Genes were subsequently filtered according to differential stability to retain those showing reproducible spatial expression patterns across donors. To address the sparse right-hemisphere sampling of the AHBA, left–right mirroring was used to generate bilateral regional expression profiles, and missing regional values were interpolated where necessary. The final region-by-gene expression matrix was aligned with the BNA-246 regional imaging phenotype for subsequent PLS analysis.

## Gene enrichment analysis

Functional annotation of genes showing spatial associations with the neuroimaging phenotype was performed using the XianTao platform (https://www.xiantaozi.com/). Gene Ontology (GO) and Kyoto Encyclopedia of Genes and Genomes (KEGG) enrichment analyses were conducted to identify overrepresented biological functions and pathways, with an FDR-corrected P value < 0.05 considered statistically significant. The top 10 significant terms from each analysis were selected for visualization. Organ-, tissue-, and cell-type-specific expression patterns were further assessed using WebCSEA (https://bioinfo.uth.edu/webcsea/). Brain-region- and developmental-stage-specific expression enrichment was evaluated using the CSEA tool (http://doughertytools.wustl.edu/CSEAtool.html).

# Supplementary Tables

**Table S1. Between-study heterogeneity and assessment of small-study effects for brain regions identified in the meta-analysis.**

|  | Regions | *I^2^* (%) | Egger’s *p* value | Begg’s *p* value |
| --- | --- | --- | --- | --- |
| DR>HC | Left cerebellum | 29.8 | 0.767 | 1.000 |
|  | Corpus callosum | 24.0 | 0.125 | 1.000 |
| DR<HC | Left calcarine fissure | 6.9 | 1.000 | 1.000 |
|  | Left middle occipital gyrus | 3.7 | 0.401 | 1.000 |
|  | Right postcentral gyrus | 1.7 | 0.263 | 1.000 |
|  | Right anterior cingulate | 1.8 | 1.000 | 1.000 |
|  | Right postcentral gyrus | 5.9 | 0.013 | 1.000 |
|  | Right middle temporal gyrus | 0.8 | 1.000 | 1.000 |
| Abbreviations: DR, patients with diabetic retinopathy; HC, healthy controls. P values for Egger’s and Begg’s tests were adjusted for multiple comparisons using the Holm method. | | | | |

**Table S2. Jackknife sensitivity analyses of the brain regions with increased resting-state function in DR.**

| Study omitted | Left cerebellar  (-14, -74, -42) | Corpus callosum  (14, -66, 40) |
| --- | --- | --- |
| Wang et al.^(23^) | 1 | 0 |
| Qi et al.^(21)^ | 1 | 1 |
| Shi et al.^(22)^ | 1 | 1 |
| Zhong et al.^(24)^ | 1 | 1 |
| Zhong et al.^(43)^ | 1 | 1 |
| Wang et al.^(40)^ | 1 | 1 |
| Chen et al.^(48)^ | 1 | 0 |
| Huang et al.^(41)^ | 1 | 1 |
| Li et al. ^(45)^ | 1 | 1 |
| Qi et al.^(46)^ | 1 | 1 |
| Liao et al.^(44)^ | 0 | 1 |
| Wang et al.^(42)^ | 1 | 1 |
| Yu et al.^(47)^ | 1 | 1 |
| Total | 12 | 11 |

**Table S3. Jackknife sensitivity analyses of the brain regions with decreased resting-state function in DR.**

| Study omitted | Left calcarine fissure  (-2, -68, 14) | Right postcentral gyrus  (62, -14, 18) | Right anterior cingulate  (4, 44, 4) | Left middle occipital gyrus  (-20, -98, 4) | Right postcentral gyrus  (32, -34, 54) | Right middle temporal gyrus  (54, -8, -24) |
| --- | --- | --- | --- | --- | --- | --- |
| Wang et al.^(23^) | 1 | 1 | 1 | 1 | 1 | 1 |
| Qi et al.^(21)^ | 1 | 1 | 1 | 1 | 1 | 1 |
| Shi et al.^(22)^ | 1 | 1 | 1 | 1 | 1 | 1 |
| Zhong et al.^(24)^ | 1 | 1 | 1 | 1 | 1 | 1 |
| Zhong et al.^(43)^ | 1 | 1 | 1 | 1 | 1 | 1 |
| Wang et al.^(40)^ | 1 | 1 | 1 | 1 | 1 | 0 |
| Chen et al.^(48)^ | 1 | 1 | 1 | 1 | 1 | 1 |
| Huang et al.^(41)^ | 1 | 1 | 1 | 1 | 1 | 1 |
| Li et al. ^(45)^ | 1 | 1 | 1 | 1 | 1 | 1 |
| Qi et al.^(46)^ | 1 | 1 | 1 | 1 | 1 | 1 |
| Liao et al.^(44)^ | 1 | 1 | 1 | 1 | 1 | 1 |
| Wang et al.^(42)^ | 1 | 1 | 1 | 1 | 1 | 0 |
| Yu et al.^(47)^ | 1 | 1 | 1 | 1 | 1 | 1 |
| Total | 13 | 13 | 13 | 13 | 13 | 11 |

**Table S4. Leave-one-modality-out analyses of the brain regions with increased resting-state function in DR.**

| Excluded modality | Left cerebellar  (-14, -74, -42) | Corpus callosum  (14, -66, 40) |
| --- | --- | --- |
| ALFF | 1 | 0 |
| ASL | 1 | 1 |
| DC | 1 | 1 |
| VMHC | 1 | 1 |
| dFCD | 1 | 1 |
| ReHo | 0 | 1 |
| FC | 1 | 0 |
| Total | 6 | 5 |

**Table S5. Leave-one-modality-out analyses of the brain regions with decreased resting-state function in DR.**

| Excluded modality | Left calcarine fissure  (-2, -68, 14) | Left middle occipital gyrus  (-20, -98, 4) | Right postcentral gyrus  (62, -14, 18) | Right anterior cingulate  (4, 44, 4) | Right postcentral gyrus  (32, -34, 54) | Right middle temporal gyrus  (54, -8, -24) |
| --- | --- | --- | --- | --- | --- | --- |
| ALFF | 1 | 1 | 1 | 1 | 1 | 1 |
| ASL | 1 | 1 | 1 | 1 | 1 | 1 |
| DC | 1 | 1 | 1 | 1 | 1 | 0 |
| VMHC | 1 | 1 | 1 | 1 | 1 | 0 |
| dFCD | 1 | 1 | 1 | 1 | 1 | 1 |
| ReHo | 1 | 1 | 1 | 1 | 1 | 1 |
| FC | 1 | 1 | 1 | 1 | 1 | 1 |
| Total | 7 | 7 | 7 | 7 | 7 | 5 |

# Supplementary Figures

## Figure S1. Funnel plots for the principal meta-analytic findings.

Each point represents the study-specific effect size at the corresponding SDM peak.

# Supplementary references

Albajes-Eizagirre, A., et al.

2019	Voxel-based meta-analysis via permutation of subject images (PSI): Theory and implementation for SDM. Neuroimage 186:174-184.

Baiano, M., et al.

2007	Anterior cingulate volumes in schizophrenia: a systematic review and a meta-analysis of MRI studies. Schizophr Res 93(1-3):1-12.

Cai, M., et al.

2024	Homotopic functional connectivity disruptions in schizophrenia and their associated gene expression. Neuroimage 289:120551.

Medisauskaite, A., and C. Kamau

2017	Prevalence of oncologists in distress: Systematic review and meta-analysis. Psychooncology 26(11):1732-1740.
